# Antibiotic potentiating effect of *Bauhinia purpurea* L. against multidrug resistant *Staphylococcus aureus*

**DOI:** 10.3389/fmicb.2024.1385268

**Published:** 2024-04-16

**Authors:** Kundhey Hang Limboo, Bimala Singh

**Affiliations:** Department of Microbiology, School of Life Sciences, Sikkim University, Gangtok, Sikkim, India

**Keywords:** multidrug resistance, *Staphylococcus aureus*, *Bauhinia purpurea* L., antibiotic potentiation, synergistic activity

## Abstract

*Bauhinia purpurea* L. is a medium-sized tree from the family *Fabaceae*. The plant is traditionally used as medicine by different tribes in Sikkim. The present study aimed to evaluate the modulation in minimum inhibitory concentration (MIC) of the bark methanol extract of *Bauhinia purpurea* L. against the clinical isolates of multidrug resistant *Staphylococcus aureus*. The synergistic activity of the test plant extract with different classes of antibiotics was also evaluated. The methanol extract of *Bauhinia purpurea* exhibited modulation by a 16-fold reduction in the MIC of clindamycin against both resistant and susceptible isolates, followed by penicillin and gentamicin, whereas a maximum of only a 4-fold MIC reduction was observed with ciprofloxacin. The lowest minimum inhibitory concentration and minimum bactericidal concentration showed by the plant extract was 0.48 and 0.97 mg/mL, respectively. The methanol extract of *Bauhinia purpurea* exhibited synergistic activity with penicillin, gentamicin, ciprofloxacin, and clindamycin against most of the tested isolates of multidrug-resistant *Staphylococcus aureus* (MDR-SA). Gas chromatography-mass spectrometry analysis of *Bauhinia purpurea* L. bark methanol extract revealed 16 phytocompounds. The results provide an insight into the potential antibacterial property of the plant extract in terms of its antibiotic MIC modulation and synergistic properties with the selected antibiotics. This is the first report of the antibiotic potentiation property of *Bauhinia purpurea* L., collected from Sikkim, India.

## Introduction

*Staphylococcus aureus* is a human pathogen related to a plethora of infections, from folliculitis, and furunculosis to adverse conditions such as sepsis, deep abscesses, osteomyelitis, bloodstream infections, and infective endocarditis (van Belkum et al., 2009). These infections could lead to a rise in morbidity as well as mortality rates (Turner et al., 2019). *S. aureus* resistant to antibiotic oxacillin is usually methicillin-resistant *S. aureus* (MRSA; Lee et al., 2018) and falls under the category of “Priority 2: High” of the World Health Organization's (WHO) priority pathogens list (World Health Organization, 2017) for development of drugs. *S. aureus* has evolved a multidrug resistance profile, causing hindrance to treatment options. The emergence of multidrug-resistant *S. aureus* (MDR-SA) has become much distressing due to its resistance to an increased number of antibiotic classes, including methicillin, vancomycin (glycopeptide), daptomycin (lipopeptide), linezolid (oxazolidinone), tedizolid (anoxazolidinone), dalbavancin (lipoglycopeptide), oritavancin (glycopeptide), ceftaroline (β-lactam antibiotic), ceftobiprole, and carbapenems (Gatadi et al., 2019). Resistance of *S. aureus* to antibiotics is due to diverse mechanisms that include reduced impermeability of the antibiotics through the cell membrane, the formation of biofilm, altered drug targets, efflux pumps, enzyme expression inhibition, and transfer of drug-resistant plasmid (Roberts et al., 2012). Thus, the discovery and development of novel antibacterial agents are urgent needs currently.

Plants have been an active ingredient in traditional medicine systems since ancient times and can be studied as effective models for designing antimicrobial agents to combat drug resistant bacteria (Petrovska, 2012). Secondary metabolites acquired from plants can be used to uncover potentially strong, non-toxic, and low-cost medicines, as the World Health Organization supports scientifically proven traditional medicines (World Health Organization, 2013). Studies on various plant extracts and antibiotics have reported synergistic antibacterial activity against antibiotic resistant bacteria (Chandra et al., 2017). These combination approaches have an advantage over synthetic antimicrobial agents in terms of their reduced side effects, increased efficiency, increased stability and bioavailability, and the need for lower doses in comparison to synthetic drugs (Cheesman et al., 2017). Moreover, restoring the activity of conventional antibiotics using combinations would enable the drug to reach clinical usage much more rapidly. Therefore, the plant extracts or pure compounds can be merged with conventional antibiotics to explore new antimicrobials for the treatment of infectious diseases (Abreu et al., 2017). The plant extracts can be used as adjuvant drugs to amplify the antimicrobial activity of conventional antibiotics (Bao et al., 2020).

Sikkim is a north-eastern state of India bestowed with a flora of abundant diversity, including medicinal plants (ENVIS, 2020). The medicinal plants found in Sikkim are used by the local population for the treatment of various infections and illnesses (Singh et al., 2002). *Bauhinia purpurea* L., commonly known as the orchid tree, is a medium-sized tree used in folk medicine by the indigenous Limboo tribes of Sikkim in the Kanchendzonga Bioreserve Region (Badola and Pradhan, 2013). Its medicinal applications range from controlling diarrhea with bark extract to the laxative action of flowers and the carminative effect of roots. The bark, root, and flowers are also useful as maturant for boils and abscesses. It is also used against animal bite as per the ethnobotanical survey in Sikkim (Das et al., 2012). Some major phytoconstituents present in the stems and leaves of *B. purpurea* include alkaloids, saponins, phenols, flavonoids, fats, anthocyanins, terpenoids, and steroids (Krishnaveni, 2015). Anti-inflammatory, anti-arthritic, and analgesic activities of *B. purpurea* have been reported (Shreedhara et al., 2009; Kumar et al., 2019). A dose of 5,000 mg/kg *B. purpurea* methanol leaf extracts showed no toxic effects in rats (Zakaria et al., 2011). This is the first report of the antibiotic potentiation property of *Bauhinia purpurea* L., collected from Sikkim, India.

## Materials and methods

All the chemicals, reagents, and culture media were acquired from HiMedia Laboratories Private Limited, India, and Merck, Germany.

### Plant collection

The bark of *B. purpurea* L. was collected in April 2022 from Geyzing, West Sikkim, India (27°17′166′'N, 88°14′111′'E). The herbarium was prepared, and the specimen was identified by the taxonomist at the University of North Bengal, India. The voucher specimen of *B. purpurea* L. (Accession No. 12636) was deposited at the Plant Taxonomy Division, Department of Botany, University of North Bengal, Siliguri, West Bengal, India.

### Plant extract preparation

The bark of the plant was collected, washed thoroughly, and shade-air-dried for 20–25 days. The dried bark was chopped into small pieces, and pulverized using a blender (Bajaj, GX 11 750W), and further sieved to obtain a fine powder. Methanol extract was prepared from 10 g of powder mixed with 100 ml of methanol for 16 h using a Soxhlet extractor. The temperature of the heating mantle was set at 50°C. The extract was then filtered using Whatman No. 1 filter paper and dried using a rotary evaporator (Equitron). The extract was stored in vials at 4°C until future use (Seidel, 2005).

### Test microorganism

Twenty clinical isolates of *S. aureus* were procured from Sir Thutob Namgyal Memorial, Multi-Specialty Hospital, Sochakgang, Gangtok, India. The isolates were initially cultured in Mannitol Salt Agar to purify and avoid any mixed culture. Subsequently, the isolates were cultured and maintained in Tryptic Soy Agar plates and kept at 4°C (Missiakas and Schneewind, 2013). Colony morphology examination, Gram staining, catalase test, coagulase test, mannitol fermentation test, and DNase test were performed for the phenotypic identification of clinical isolates.

### Molecular identification of the clinical isolates by 16S rRNA gene sequencing

The Wizard^TM^ DNA Purification Kit (Promega) was used to extract the genomic DNA from the fresh, pure colonies of *S. aureus* as per the manufacturer's specifications. The universal primers 27 F 5′-AGAGTTTGATCCTGGCTCAG-3′ and 1492 R 5′-GGTTACCTTGTTACGACTT-3′ were used for DNA amplification. The amplified DNA fragments were examined by agarose gel electrophoresis and observed with the Gel Doc^TM^ EZ Imager (Biorad). The 16S rDNA gene sequences were manually checked and analyzed using the software BioEdit, Version 7.2.6.1 (Tom Hall, Ibis Biosciences, Carlsbad, California, USA). The sequences obtained after analysis were compared to the NCBI (National Center for Biotechnology Information) online database using BLAST (Basic Local Alignment Search Tool) to retrieve similar sequences.

### Antimicrobial susceptibility testing

The antibiotic resistance profile of the clinical isolates was determined by the Kirby-Bauer disk diffusion method following the CLSI guidelines (Hudzicki, 2009; Clinical Laboratory Standard Institute, 2020). Antibiotic susceptibility test was performed using antibiotic discs belonging to nine antimicrobial categories, including Penicillin (10 U; Penicillinase-labile penicillin), Cefoxitin (30 μg; Penicillinase-stable penicillin), Gentamicin (10 μg), Amikacin (30 μg), Kanamycin (30 μg; Aminoglycosides), Erythromycin (15 μg; Macrolides), Tetracycline (30 μg; Tetracyclines), Ciprofloxacin (5 μg), Norfloxacin (10 μg), Ofloxacin (5 μg; Fluoroquinolones), Clindamycin (2 μg; Lincosamides), Trimethoprim (5 μg; Folate pathway antagonists), and Chloramphenicol (30 μg; Phenicols). *S. aureus* (MTCC 740) was used as the antibiotic sensitive control.

### Screening for methicillin resistance

For selective isolation of methicillin-resistant *S. aureus*, clinical isolates were cultured in Methicillin-resistant *S. aureus* agar (MeReSa) plates. The isolates were also tested for susceptibility to methicillin using a cefoxitin (30 μg) disc. Results were interpreted in compliance with the standard established by the CLSI guidelines (Clinical Laboratory Standard Institute, 2020).

### Screening for vancomycin resistance

Vancomycin resistance was determined using the method given by Moosavian et al. (2020). Brain Heart Infusion (BHI) agar containing vancomycin (6 μg/ml) and 4% NaCl was prepared. Then, 10 μL of inoculum at OD of 0.1 corresponding to 0.5 McFarland standard was inoculated onto the agar surface. The plates were then incubated at 37°C for 24 h. Growth after 24 h of incubation in the medium would be considered as vancomycin-resistant isolates (Clinical Laboratory Standard Institute, 2020).

### Determination of antibacterial activity of plant extract

Antibacterial activity was determined by the agar well diffusion method using Mueller Hinton Agar (MHA) plates (Perez, 1990). The *S. aureus* isolates were cultured overnight in Tryptic Soy Broth (TSB) at 37°C. Meanwhile, the plant extract concentration was prepared at 500 mg/mL in 10% dimethyl sulfoxide (DMSO). The OD of the overnight bacterial culture was measured in spectrophotometer and adjusted to 0.1 at 625 nm corresponding to 0.5 McFarland Standard. 100 μl of inoculum was spread onto the MHA agar plate. Three wells were bored using 8 mm sterile cork-borer. 100 μl of plant extract, antibiotic, and DMSO were added to each well. The plates were incubated at 37°C for 24 h. Vancomycin (30 μg/ml) was used as the antibiotic positive control (Periasamy et al., 2019). DMSO was used as the negative control (Gonelimali et al., 2018). Antibacterial activity was determined by measuring the diameter of the inhibition zone, inclusive of the well diameter of 8 mm. All the experiments were carried out in triplicate. The data was represented as mean values ± SD.

### Determination of MIC and MBC

The minimum inhibitory concentration (MIC) of the bark methanol extract of *B. purpurea* and antibiotics, viz., penicillin (β-lactam class), gentamicin (aminoglycosides), ciprofloxacin (fluoroquinolones), and clindamycin (lincosamides), were determined by the broth microdilution method in a 96 well microtitre plate against all the clinical isolates of *S. aureus* (Elshikh et al., 2016). Firstly, 100 μl of the 250 mg/mL plant extract/MHB broth was dispensed in each well of Column 1 of the 96-well microtitre plate. Then columns 2–10 were filled with 50 μl of cation adjusted MHB broth only. Column 11 contained 100 μl of diluted standardized inoculum, and Column 12 contained 100 μl of medium broth only (media sterility control). A multichannel pipette was then used to transfer and mix plant extracts from columns 1–10, resulting in 50 μl of plant extract per well. The test concentrations of the different plant extracts achieved through double serial dilutions from columns 10–1 ranged from 250 to 0.48 mg/mL. A similar procedure was followed for different antibiotics, but the concentration of antibiotics ranges from 500 to 0.97 μg/ml. The microorganism suspension with OD at 625 nm corresponding to 0.5 McFarland Standard was then diluted in MHB broth (1:100). 50 μl of the diluted bacterial suspension was then added to all wells containing plant extract, resulting in ~5 × 10^5^ CFU/mL of bacterial suspension. The time taken to prepare and dispense the OD adjusted bacteria did not exceed 15 min. After incubation for 24 h at 37°C, resazurin (0.015 %) was added to all wells (30 μl per well) and further incubated for 2 h for the observation of color change. After incubation, columns showing no color change of resazurin indicated the absence of bacterial growth and were scored as above the MIC value. The minimum bactericidal concentration (MBC) of plant extract was determined by plating directly the content of wells with concentrations higher than the MIC value. The MBC value was determined when there was no colony growth from the directly plated contents of the wells after 24 h of incubation at 37°C. All experiments were done in triplicate.

### Determination of synergistic activity of *B. purpurea* bark methanol extract with antibiotics

The MIC modulator effect was determined by the synergistic activity of *B. purpurea* bark methanol extract with different combinations of antibiotics. It was evaluated by the checkerboard microdilution assay using a 96-well microtitre plate as described by Bellio et al. (2015) with some modifications. Firstly, 50 μl of microbial growth medium (Mueller Hinton broth) were added to each microplate well. In column 1, 50 μl of the desired antibiotic concentration was added. From columns 1–6, a 2-fold dilution was made using a multichannel pipette. To row A, a 50-μl plant extract aliquot was added and serially diluted until row G. Row H contained only the antibiotic. Column 7 contained only the plant extract. The bacterial suspension with OD at 625 nm corresponding to 0.5 McFarland Standard was then diluted in MHB broth (1:100). 50 μl of the diluted bacterial suspension was then added to all tested wells. Column 8 contained only inoculum as the growth control, whereas column 9 was the medium sterility control. The microtitre plates were incubated for 24 h at 37°C. Finally, 30 μl of freshly prepared 0.015% resazurin was added to all wells and further incubated for 2 h.

The synergistic effect was evaluated by the calculation of the Fractional Inhibitory Concentration (FIC) Index. Synergy was indicated by the FIC index value of ≤ 0.5, > 0.5 to ≤ 1 as additive; > 1 to ≤ 4 as indifferent, and >4 as antagonistic (Saiman, 2007).

### Minimum inhibitory concentration reversal

The ability of *B. purpurea* methanol bark extract to reverse the MIC of antibiotics was determined by fold-reduction in MIC and represented as the modulation factor (MF) calculated as MIC_Drug_/MIC_Drug_ in combination (Christena et al., 2015).

### GC-MS analysis

The phytoconstituents of *B. purpurea* bark methanol extract was analyzed by Gas Chromatography-Mass Spectrometry (GC-MS; QP 2010 Ultra Shimadzu). The headspace sampler (AOC-20s) and auto-injector (AOC-20i), equipped with a mass selective detector, have an ion source temperature of 220°C, an interface temperature of 270°C, a solvent cut time of 2.50 min, a threshold of 1,000 eV, and a mass range of 40–650 m/z. Carrier gas was helium at a linear velocity of 40.5 cm/s with a 10:0 split ratio. The oven temperature was set at 80°C, the rise rate was up to 10°C/min with a 3-min hold, and then 280°C with a 19-min hold. The temperature of the injector was maintained at 270°C, and the volume of samples injected was 1 μl. Compounds present in the plant extracts tested were identified by comparing their mass spectra with those present in the National Institute of Standards and Technology (NIST14) and WILEY8 libraries.

## Results

### Identification of clinical isolates

**Figure 1:**
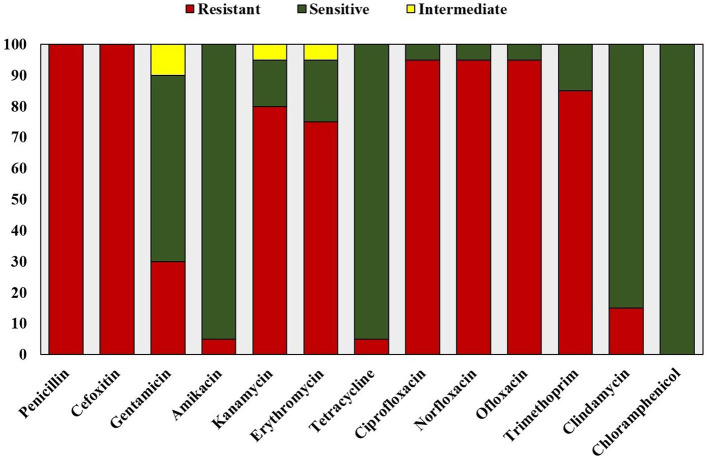
Antibiotic susceptibility test profile of clinical isolates of *Staphylococcus aureus*.

**Figure 2:**
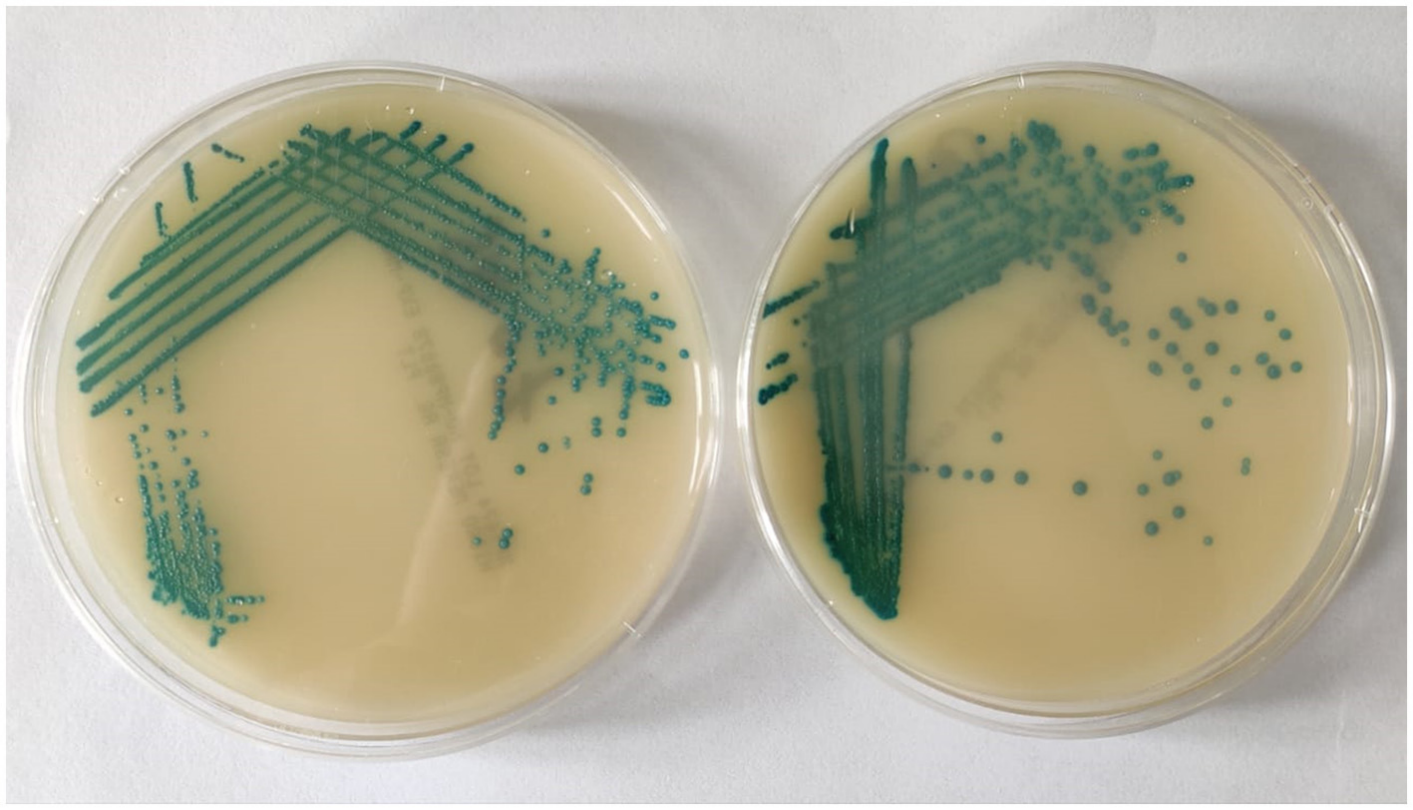
Methicillin resistant *Staphylococcus aureus* in MeReSa agar plates.

All the clinical isolates were confirmed to be *Staphylococcus aureus* by phenotypic and genotypic identification. The GenBank accession numbers for the 16S rRNA gene sequence of the clinical isolates of *Staphylococcus aureus* are shown in Supplementary Table 1. All the isolates were resistant to at least one agent in three or more antibacterial categories (Magiorakos et al., 2012). Hence, they are identified as multidrug resistant *S. aureus* on the basis of disk diffusion antibiotic susceptibility test results and the zone diameter breakpoints mentioned in Clinical Laboratory Standard Institute (2020; Figure 1). All the isolates were resistant to methicillin (Figure 2). Vancomycin-resistant strains were absent during the screening.

### Determination of antibacterial activity

**Figure 3:**
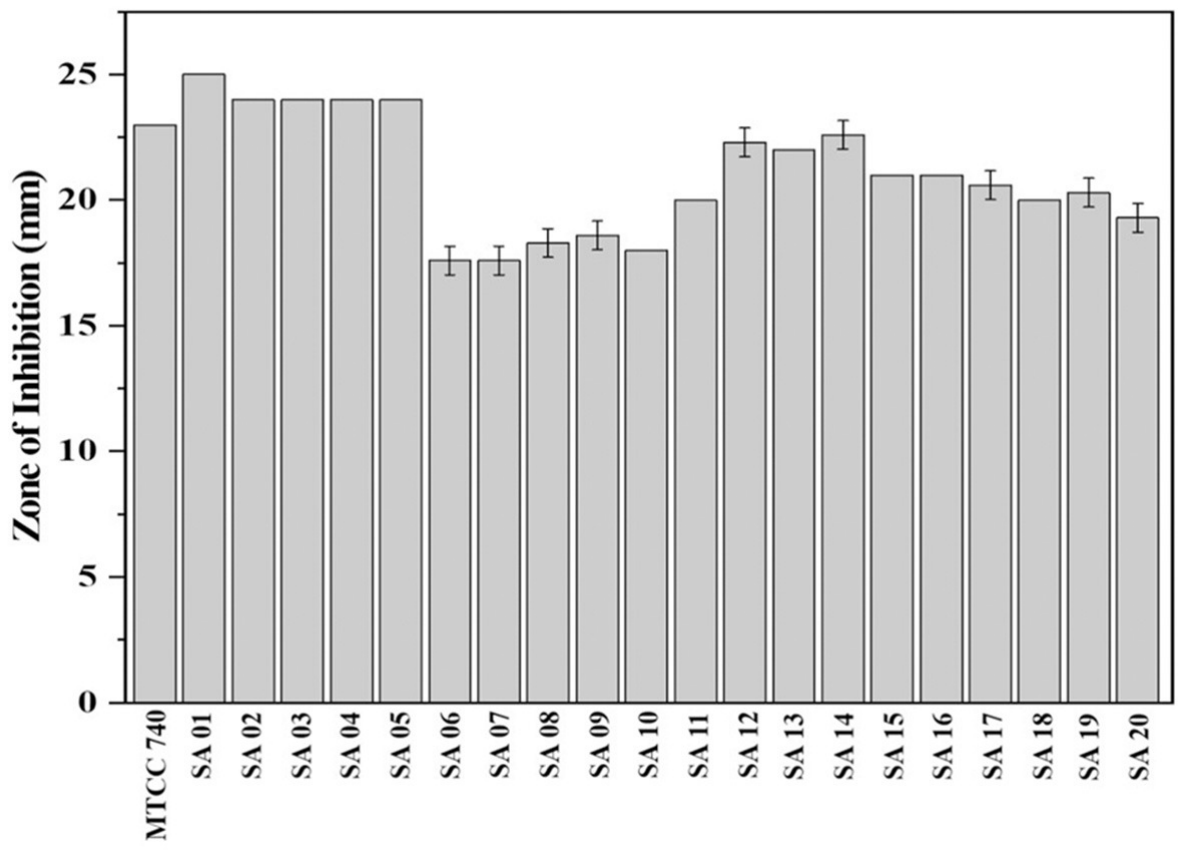
Zone of inhibition of *Bauhinia purpurea* L. bark methanol extract against isolates of MDR *Staphylococcus aureus*.

The methanol bark extract of *B. purpurea* exhibited an antibacterial effect with an inhibition zone ranging from 17.6 ± 0.57 to 25 ± 0.0 mm against the clinical isolates of MDR *S. aureus* and the antibiotic sensitive control *S. aureus* (MTCC 740), as shown in Figure 3. The largest zone of inhibition was 25 ± 0.0 mm observed for isolate SA 01.

### Determination of MIC and MBC

The lowest MIC of *B. purpurea* bark methanol extract was 0.48 mg/mL, and MBC was 0.97 mg/mL observed for isolate SA 08. The highest MIC and MBC were 7.81 and 31.25 mg/mL, respectively, for isolate SA 07. The ratio of MBC/MIC was ≤ 4 (Table 1).

**Table 1:** MIC and MBC of *Bauhinia purpurea* L. bark methanol extract against clinical isolates of *Staphylococcus aureus*.

**Sl. No**.	**Isolate**	**MIC (mg/mL)**	**MBC (mg/mL)**	**MBC/MIC ratio**
01	SA 01	1.95	3.90	2
02	SA 02	3.90	7.81	2.002
03	SA 03	1.95	3.90	2
04	SA 04	1.95	3.90	2
05	SA 05	0.97	1.95	2.010
06	SA 06	0.97	1.95	2.010
07	SA 07	7.81	31.25	4.001
08	SA 08	0.48	0.97	2.020
09	SA 09	0.97	3.90	4.020
10	SA 10	0.97	1.95	2.010
11	SA 11	3.90	15.62	4.005
12	SA 12	3.90	15.62	4.005
13	SA 13	1.95	7.81	4.005
14	SA 14	1.95	7.81	4.005
15	SA 15	1.95	7.81	4.005
16	SA 16	1.95	7.81	4.005
17	SA 17	1.95	7.81	4.005
18	SA 18	1.95	7.81	4.005
19	SA 19	1.95	7.81	4.005
20	SA 20	1.95	7.81	4.005
21	*S. aureus* (MTCC 740)	1.95	3.90	2

### Determination of synergistic activity of *B. purpurea* bark methanol extract with antibiotics

A synergistic effect was observed with *B. purpurea* bark methanol extract with antibiotics, namely penicillin, gentamicin, and clindamycin, against clinical isolates of MDR *S. aureus*. This was determined by the calculation of FIC indices, which ranged from 0.12 to 0.5 (Table 2). A modulation in the MIC of the tested plant extract was exhibited by a decrease in the MIC of the respective antibiotics in combination with the plant extract when compared with the MIC of the antibiotic alone. The MIC value of penicillin was 125 μg/ml against isolate SA 07 and SA 08, which was reduced to 16-fold (7.81 μg/ml) when combined with *B. purpurea* bark methanol extract. Similarly, the MIC value of penicillin was also reduced from 62.5 to 3.90 μg/ml when combined with the plant extract for isolate SA 04. However, for the isolates SA 01 and SA 02, the FIC index of the plant extract when combined with penicillin was equal to 1, thereby exhibiting an additive effect. The MIC of the methanol extract of *B. purpurea* in combination with clindamycin was 0.01 μg/ml against nine isolates. These findings revealed the synergistic properties of *B. purpurea* bark methanol extract with the antibiotics penicillin, gentamicin, and clindamycin, thereby inhibiting the growth of the clinical isolates. However, the extract showed synergistic, additive, and indifferent effects when combined with ciprofloxacin.

**Table 2:** Determination of synergistic activity of *B. purpurea* bark methanol extract with antibiotics.

**Isolates**	**Penicillin**			**Gentamicin**			**Ciprofloxacin**			**Clindamycin**		
	**MIC1** ^*^	**MIC2** ^**^	Σ**FIC**	**MIC1**	**MIC2**	Σ**FIC**	**MIC1**	**MIC2**	Σ**FIC**	**MIC1**	**MIC2**	Σ**FIC**
SA 01	250	125	1.00	15.62	3.90	0.48	15.62	15.62	2.00	0.48	0.03	0.12
SA 02	15.62	7.81	1.00	0.97	0.06	0.12	3.90	1.95	1.00	0.48	0.03	0.12
SA 03	125	31.25	0.50	7.81	1.95	0.48	15.62	15.62	2.00	0.48	0.03	0.12
SA 04	62.50	3.90	0.12	7.81	1.95	0.48	15.62	7.81	1.00	0.48	0.03	0.12
SA 05	31.25	3.90	0.24	31.25	15.62	1.00	31.25	31.25	2.00	0.48	0.03	0.12
SA 06	250	15.62	0.12	3.90	1.95	1.00	31.25	15.62	1.00	0.48	0.03	0.12
SA 07	125	7.81	0.12	15.62	0.97	0.12	31.25	7.81	0.50	0.48	0.03	0.12
SA 08	125	7.81	0.12	15.62	7.81	1.00	31.25	15.62	1.00	0.48	0.03	0.12
SA 09	250	15.62	0.12	62.5	15.62	0.48	31.25	15.62	1.00	0.48	0.03	0.12
SA 10	250	15.62	0.12	7.81	1.95	0.48	31.25	15.62	1.00	0.48	0.03	0.12
SA 11	250	15.62	0.12	62.5	7.81	0.24	31.25	7.81	0.50	0.12	0.01	0.20
SA 12	125	15.62	0.24	31.25	3.90	0.24	31.25	7.81	0.50	0.12	0.01	0.20
SA 13	125	15.62	0.24	15.62	1.95	0.24	31.25	7.81	0.50	0.12	0.03	0.50
SA 14	125	31.25	0.50	31.25	7.81	0.50	31.25	7.81	0.50	0.12	0.03	0.50
SA 15	62.5	7.81	0.24	0.97	0.12	0.24	31.25	7.81	0.50	0.12	0.03	0.50
SA 16	125	31.25	0.50	62.5	7.81	0.24	15.62	3.90	0.50	0.12	0.03	0.50
SA 17	125	31.25	0.50	62.5	15.62	0.50	31.25	7.81	0.50	0.12	0.03	0.50
SA 18	125	31.25	0.50	31.25	7.81	0.24	31.25	7.81	0.50	0.12	0.03	0.50
SA 19	31.25	3.90	0.24	125	15.62	0.24	31.25	7.81	0.50	250	62.5	0.50
SA 20	62.5	15.62	0.50	62.5	7.81	0.24	31.25	7.81	0.50	0.12	0.03	0.50
MTCC 740 *S. aureus*	1.97	0.12	0.12	0.01	0.005	1.00	0.48	0.06	0.24	0.48	0.03	0.12

^*^MIC1, MIC of antibiotic alone; MIC2, MIC of antibiotic in combination with *B. purpurea* methanol extract.

^**^ΣFIC: Synergistic ≤ 0.5, > 0.5 to ≤ 1 additive; > 1 to ≤ 4 indifferent; > 4 antagonistic (Saiman, 2007).

### Characterization of *B. purpurea* bark methanol extract by GC-MS

GC-MS analysis of *B. purpurea* L. bark methanol extract revealed 16 compounds. The various phytocomponents detected are shown in Table 3. The highest peak area percentage was observed for 1-Dodecanol with 41.30%, followed by Flavone 4′-OH,5-OH,7-di O-glucosid with 23.58 %, and 1-Tetradecanol, acrylate with 15.48%.

**Table 3:** Phytocomponents detected by GC-MS analysis of *Bauhinia purpurea* L. bark methanol extract.

**Peak**	**Retention time**	**Area%**	**Compound name**	**Molecular formula**	**Molecular weight**
1	12.057	41.30	1-Dodecanol	C_12_H_26_O	186
2	13.450	0.50	2(5H)-Furanone, 5-(1-methylethyl)-	C_7_H_10_O_2_	126
3	14.348	15.48	1-tetradecanol, acrylate	C_17_H_32_O_2_	268
4	15.737	0.64	1-Nonanol	C_9_H_20_O	144
5	16.726	0.94	Nonanoic acid, 7-methyl-methyl ester	C_11_H_22_O_2_	186
6	17.145	0.78	1,2-Benzenedicarboxylic acid, dibutyl ester	C_16_H_22_O_4_	278
7	17.664	3.51	Propanoic acid, 3-mercapto-, dodecyl ester	C_15_H_30_O_2_S	274
8	18.287	0.76	Dodec-4-enal	C_12_H_22_O	182
9	21.889	4.53	1,2-Benzenedicarboxylic acid	C_24_H_38_O_4_	390
10	23.926	0.48	Succinic acid, di(geranyl)ester	C_24_H_38_O_4_	390
11	25.433	1.86	Hexadecanoic acid, tetradecyl ester	C_30_H_60_O_2_	452
12	27.774	1.13	Pentadecanal-	C_15_H_30_O	226
13	31.247	1.91	2H-Pyran, 2-(7-heptadecynyloxy)tetrahydro-	C_22_H_40_O_2_	336
14	32.450	2.20	1,1,4,7-Tetramethyldecahydro-1H-cyclopropa[e]azulen-4-ol	C_15_H_26_O	222
15	35.336	0.39	12-Heptadecyn-1-ol	C_17_H_32_O	252
16	36.984	23.58	Flavone 4′-OH,5-OH,7-di-O-glucoside	C_27_H_30_O_15_	294

## Discussion

Antibiotic resistance has created a bottleneck in therapeutic options, escalating health care costs, extended hospital stays, and a high risk of death in both developed and developing countries (Schwaber and Carmeli, 2007; Cornejo-Juárez et al., 2015; Gulen et al., 2015; Founou et al., 2017). Plant extracts or bioactive compounds, when combined with antibiotics, have exhibited reduced antibiotic resistance in drug resistant bacteria (Su et al., 2020; Chawla et al., 2022). The antibacterial property of *Bauhinia purpurea* is well-documented (Negi et al., 2012), but limited knowledge is available regarding its antibiotic potentiation effects with resistant antibiotics against clinical isolates of *Staphylococcus aureus*. Thus, the present study has evaluated the antibacterial activity as well as the antibiotic potentiation effect of the methanol extract of *B. purpurea* against 20 clinical isolates of MDR *S. aureus*. Antibiotic potentiation effect was determined by evaluation of synergistic activity of plant extract in combination with different classes of antibiotics along with MIC modulation of antibiotics against the clinical isolates. Following the antibacterial screening, the lowest MIC and MBC values recorded against the MDR-SA isolate were 0.48 and 0.97 mg/mL, respectively, for isolate SA 08. The MIC value of the leaf extract of *B. purpurea* against *S. aureus* (ATCC 25923) has been reported as 150 mg/mL in an earlier study (Negi et al., 2012). The MBC/MIC ratio which indicated the MIC Index value ranged from 2 to 4 in our study thus representing bactericidal effect of *B. purpurea* bark methanol extract against the tested isolates (Mogana et al., 2020). A recent study indicated that aqueous extracts of Triphala (pericarp extracts of *Terminalia chebula, Terminalia bellirica*, and *Phyllanthus emblica*) showed synergistic activity with gentamicin against some multidrug-resistant Gram-negative bacilli and with oxacillin against MRSA isolates (Manoraj et al., 2019). The present study revealed synergistic action of combination of extract and tested antibiotics.

**Figure 4:**
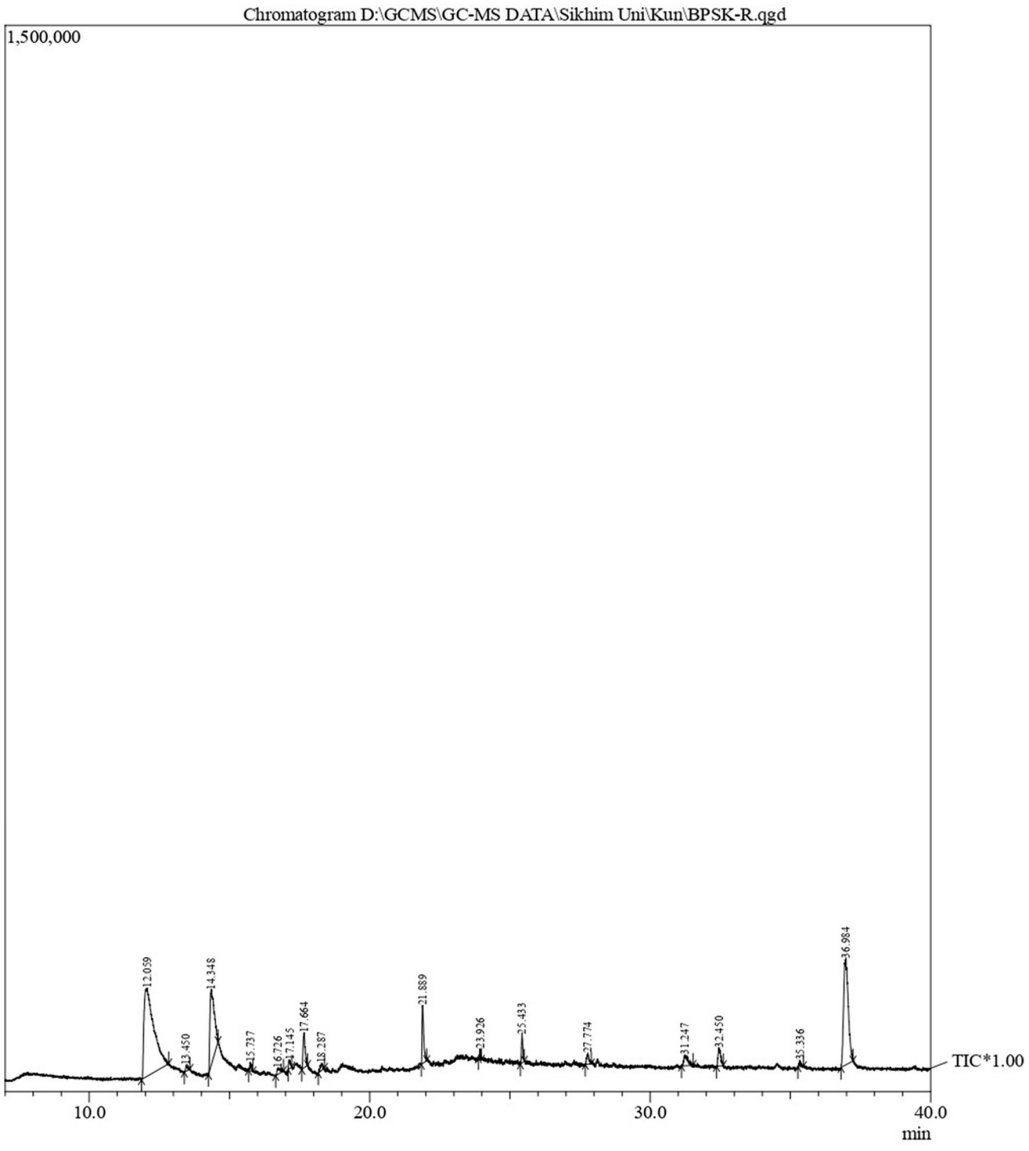
GC-MS analysis of *Bauhinia purpurea* L. bark methanol extract.

With respect to the synergistic study, the FIC index values for the combination of *B. purpurea* bark methanol extract and gentamicin for different clinical isolates ranged from 0.12 to 0.50, which indicated a synergistic effect. Similarly, the FIC values of 0.12–0.50 show a synergistic effect for the combination of *B. purpurea* methanol bark extract and penicillin for all clinical isolates of *S. aureus*. In the current study, all the clinical isolates were resistant to penicillin. The synergistic combination of penicillin and the plant extract exhibited promising antibiotic potentiation effect. The MIC value of penicillin of 250 μg/ml was reduced 16-fold to 15.62 μg/ml for four isolates. Similar synergistic reports showed MIC reduction of imipenem in combination with *Ocimum basilicum* essential oil against clinical strains of *S. aureus*, restoring β-lactam antibiotic efficacy (Bassolé et al., 2010; Araújo Silva et al., 2016). The test plant extract and ciprofloxacin exhibited synergistic effects along with additive and indifferent effects against clinical isolates. However, combinations of extract and selected antibiotics did not show any antagonistic effect. *Bauhinia purpurea* extract exhibited the maximum antibiotic potentiation effect with clindamycin exhibiting a maximum of 16-fold MIC reduction against 11 isolates (Table 4). The MIC value of clindamycin is significantly reduced to 0.01 μg/ml for nine isolates when combined with *B. purpurea* methanol extract. Similarly, MIC value of clindamycin of 0.48 μg/ml was reduced 16-fold to 0.03 μg/ml for 10 isolates. A similar reduction in MIC has been reported earlier (Vaou et al., 2021). Therefore, these results indicate the considerable antibiotic potentiation effects of the bark methanol extract of *B. purpurea* L. in terms of a reduction in the MIC of the selected antibiotics in combination with the extract along with synergistic action. It is broadly accepted that the antimicrobial activities and physiological effects of plant therapies are due to the biosynthesis of secondary metabolites produced by plants as a defense mechanism. GC-MS analysis of *B. purpurea* bark extract has revealed 16 compounds (Figure 4). The highest peak area percentage was observed for 1-Dodecanol with 41.30%, followed by Flavone 4′-OH,5-OH,7-di O-glucosid with 23.58%, and 1-Tetradecanol, acrylate with 15.48%. Among the compounds detected, 1-Dodecanal; Nonanol; 2H-Pyran,2-(7-heptadecynyloxy)tetrahydro; Propanoic acid, 3-mercapto-, dodecyl ester; Pentadecanal; 1-Tetradecanol, acrylate, and Flavone 4′-OH,5-OH,7-di-O-glucoside have also been detected in other plants exhibiting antibacterial activity against *Staphylococcus aureus* (Togashi et al., 2007; Mujeeb et al., 2014; Canli et al., 2016; Semwal et al., 2018; Gao et al., 2019; Chenniappan et al., 2020). Further studies are warranted to elucidate specific mechanisms for the antibiotic potentiation effects of the plant extract with reference to its ability to modify antibiotic target sites, modulate efflux pump activity and biofilm formation related to resistance mechanisms. However, some limitation persists in the present study with respect to molecular detection of resistant genes in the MDR isolates followed by mechanistic studies of antibiotic potentiation.

**Table 4:** Antibiotic resistance modulatory effect of *Bauhinia purpurea* L. bark methanol extract.

**Strains**	**MIC of drug/MIC of drug in combination (**μ**g/ml)**				**Modulation factor (fold reduction)**			
	PEN^*^	**GEN**	**CIP**	**CD**	**PEN**	**GEN**	**CIP**	**CD**
SA 01	250/125	15.62/3.90	15.62/15.62	0.48/0.03	2	4	1	16
SA 02	15.62/7.81	0.97/0.06	3.90/1.95	0.48/0.03	2	16	2	16
SA 03	125/31.25	7.81/1.95	15.62/15.62	0.48/0.03	4	4	1	16
SA 04	62.5/3.90	7.81/1.95	15.62/7.81	0.48/0.03	16	4	2	16
SA 05	31.25/3.90	31.25/15.62	31.25/31.25	0.48/0.03	8	2	1	16
SA 06	250/15.62	3.90/1.95	31.25/15.62	0.48/0.03	16	2	2	16
SA 07	125/7.81	15.62/0.97	31.25/7.81	0.48/0.03	16	16	4	16
SA 08	125/7.81	15.62/7.81	31.25/15.62	0.48/0.03	16	2	2	16
SA 09	250/15.62	62.5/15.62	31.25/15.62	0.48/0.03	16	4	2	16
SA 10	250/15.62	7.81/1.95	31.25/15.62	0.48/0.03	16	4	2	16
SA 11	250/15.62	62.5/7.81	31.25/7.81	0.12/0.01	16	8	4	12
SA 12	125/15.62	31.25/3.90	31.25/7.81	0.12/0.01	8	8	4	12
SA 13	125/15.62	15.62/1.95	31.25/7.81	0.12/0.01	8	8	4	12
SA 14	125/31.25	31.25/7.81	31.25/7.81	0.12/0.01	4	4	4	12
SA 15	62.5/7.81	0.97/0.12	31.25/7.81	0.12/0.01	8	8	4	12
SA 16	125/31.25	62.5/7.81	15.62/3.90	0.12/0.01	4	8	4	12
SA 17	125/31.25	62.5/15.62	31.25/7.81	0.12/0.01	4	4	4	12
SA 18	125/31.25	31.25/7.81	31.25/7.81	0.12/0.01	4	4	4	12
SA 19	31.25/3.90	125/15.62	31.25/7.81	250/62.5	8	8	4	4
SA 20	62.5/15.62	62.5/7.81	31.25/7.81	0.12/0.01	4	8	4	12
MTCC 740	1.97/0.12	0.01/0.005	0.48/0.06	0.48/0.03	16	2	8	16

^*^PEN, Penicillin; GEN, Gentamicin; CIP, Ciprofloxacin; CD, Clindamycin.

## Conclusion

The antibiotic potentiating effects of *B. purpurea* bark methanol extract against the clinical isolates of multidrug resistant *S. aureus* is well-established in the results of the present study with respect to antibiotic MIC modulation and synergistic action. Further studies are warranted on elucidating mechanisms of antibiotic potentiating properties of the bark methanol extract of *B. purpurea* L.

## Data availability statement

The datasets presented in this study can be found in online repositories. The names of the repository/repositories and accession number(s) can be found in the article/Supplementary material.

## Author contributions

KL: Data curation, Investigation, Methodology, Software, Writing – original draft, Writing – review & editing. BS: Conceptualization, Supervision, Validation, Writing – review & editing.
