# Supplementary material for: Antibiotic potentiating effect of Bauhinia purpurea L. against multidrug resistant Staphylococcus aureus
Source: Front Microbiol. 2024 Apr 16;15:1385268. doi: 10.3389/fmicb.2024.1385268 (PMC11062131; doi:10.3389/fmicb.2024.1385268)
Supplement: Supplementary file 1 [file Table_1.DOCX]

Supplementary Table 1. GenBank accession numbers for the 16S rRNA gene sequence of the clinical isolates of *Staphylococcus aureus*.

| Sl. No. | Isolate code | Accession Number |
| --- | --- | --- |
| 1 | SA 01 | PP292028 |
| 2 | SA 02 | PP292029 |
| 3 | SA 03 | PP292030 |
| 4 | SA 04 | PP292031 |
| 5 | SA 05 | PP292032 |
| 6 | SA 06 | PP292033 |
| 7 | SA 07 | PP292034 |
| 8 | SA 08 | PP292035 |
| 9 | SA 09 | PP292036 |
| 10 | SA 10 | PP292037 |
| 11 | SA 11 | PP292038 |
| 12 | SA 12 | PP292039 |
| 13 | SA 13 | PP292040 |
| 14 | SA 14 | PP292041 |
| 15 | SA 15 | PP292042 |
| 16 | SA 16 | PP292043 |
| 17 | SA 17 | PP292044 |
| 18 | SA 18 | PP292045 |
| 19 | SA 19 | PP292046 |
| 20 | SA 20 | PP292047 |
